# Advances in Photothermal Therapy for Oral Cancer

**DOI:** 10.3390/ijms26094344

**Published:** 2025-05-02

**Authors:** Jian Liang, Pei Wang, Yanfang Lin, Ao Jia, Fei Tong, Zhihua Li

**Affiliations:** 1 School of Stomatology, Jiangxi Medical College, Nanchang University, Nanchang 330006, China; liangjian673@email.ncu.edu.cn (J.L.); ndfskqyy620@ncu.edu.cn (P.W.); 413009220003@email.ncu.edu.cn (Y.L.); ndfskqyy667@ncu.edu.cn (A.J.); 2 Jiangxi Provincial Key Laboratory of Oral Diseases, Nanchang 330006, China; 3 Jiangxi Provincial Clinical Research Center for Oral Diseases, Nanchang 330006, China

**Keywords:** photothermal therapy, oral cancer, oral squamous cell carcinoma, oral potentially malignant disorders, nanomaterials

## Abstract

Oral cancer represents a critical global health issue, where traditional treatment modalities are often characterized by considerable adverse effects and suboptimal effectiveness. Photothermal therapy (PTT) offers an innovative method for tumor treatment, leveraging photothermal agents to convert light into hyperthermia, ultimately leading to tumor ablation. PTT offers unique advantages in treating oral cancer due to its superficial anatomical location and consequent accessibility to laser irradiation. PTT’s advantage is further enhanced by its capacity to facilitate drug release and promote tissue regeneration. Consequently, the application of PTT for oral cancer has garnered widespread interest and has undergone rapid development. This review outlines advances in PTT for oral cancer, emphasizing strategies to improve efficacy and combination therapy approaches. The key challenges, including temperature control and long-term biosafety, are discussed alongside future directions. The review also encompasses PTT’s role in managing oral potentially malignant disorders and postoperative defects, conditions intimately linked with oral cancer. We aim to provide guidance for emerging PTT research in oral cancer and to promote the development of precise and efficient treatment strategies.

## 1. Introduction

Oral cancer, a malignancy predominantly arising in the lip mucosa, gingiva, tongue dorsum, palate, buccal mucosa, and floor of the mouth, poses a significant global health threat, severely compromising quality of life and survival [1,2,3,4]. According to global cancer statistics, approximately 380,000 new diagnoses and 180,000 deaths occur annually, constituting 2% of global cancer-related mortality [5,6,7]. Primary risk factors encompass tobacco use, betel quid chewing, alcohol consumption, and mechanical irritation such as residual roots [8,9,10,11,12]. Notably, oral potentially malignant disorders (OPMDs), like leukoplakia and erythroplakia, exhibit morphological alterations that confer an increased risk of malignant transformation over normal mucosa and consequently warrant significant attention [13,14,15,16]. The predominant type of oral cancer, oral squamous cell carcinoma (OSCC), accounts for more than 90% of cases, and most patients present with advanced disease [17,18,19]. Frequent local invasion and regional lymph node metastasis underscore the critical need for early diagnosis and intervention [20,21,22,23,24].

The oral and maxillofacial region, essential for speech, mastication, respiration, and aesthetics, presents a therapeutic dilemma: balancing radical tumor eradication with functional preservation [25,26,27]. Current clinical paradigms rely on surgery, supplemented by adjuvant radiotherapy or chemotherapy [28,29]. However, these approaches face limitations: (1) surgical disfigurement and functional impairment [30]; (2) radiotherapy-induced complications (e.g., oral mucositis, xerostomia, osteoradionecrosis) [31]; and (3) chemotherapy-related systemic toxicity, myelosuppression, and multidrug resistance [32,33]. Consequently, despite significant advances in therapeutic procedures over recent decades, the 5-year survival rate remains consistently around 60%, and roughly one-third of treated patients experience relapse [34]. Furthermore, postoperative quality of life is greatly compromised, with studies indicating that 73.9% report dysphagia [35,36]. These challenges have spurred the development of novel strategies, including immunotherapy [37], molecular targeting [38], photodynamic therapy (PDT) [39], and photothermal therapy (PTT) [40].

PTT, an emerging modality, leverages photothermal agents (PTAs) to convert near-infrared (NIR) light into localized hyperthermia, selectively ablating heat-sensitive cancer cells while sparing healthy tissues [41,42]. Its advantages include non-invasiveness, spatiotemporal precision, repeatability, and synergistic potential with other therapies [43,44,45]. Beyond direct tumor ablation, PTT-generated heat enhances drug delivery, stimulates immunogenic antigen release, remodels the tumor microenvironment (TME), and promotes osteogenesis and soft tissue regeneration—critical for repairing post-resection maxillofacial defects [46,47,48,49]. Two factors dictate PTT efficacy: PTAs and light sources [50]. While NIR-II lasers (1000–1700 nm) address penetration depth limitations in superficial oral cancers, suboptimal PTAs performance—low photothermal conversion efficiency (PCE) and poor tumor targeting—remains a hurdle [51,52]. Recent advances in nanotechnology have yielded innovative nanomedical platforms to enhance PTAs accumulation (e.g., ligand-mediated targeting, stimuli-responsive release) and enable image-guided precision [53,54,55]. Furthermore, mild hyperthermia (40–45 °C) has been shown to stimulate osteoprogenitor proliferation and angiogenesis, facilitating post-surgical bone and soft tissue regeneration [56,57,58].

**Figure 1:**
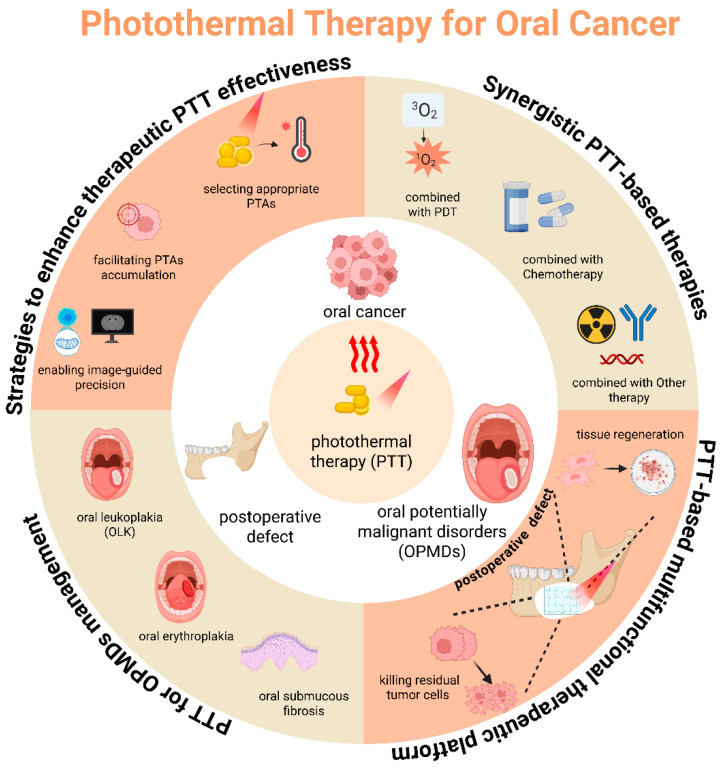
Schematic illustration of advances in photothermal therapy (PTT) for oral cancer. By leveraging photothermal agents (PTAs), PTT transforms near-infrared (NIR) light into localized hyperthermia, leading to the ablation of heat-sensitive oral cancer cells. Selecting appropriate PTAs, facilitating PTAs accumulation, and enabling image-guided precision to enhance the therapeutic effectiveness of PTT. Furthermore, when combined with other therapy modalities, PTT can exhibit synergistic effects against oral cancer. Beyond treatment, PTT can also be used for oral cancer prevention by inhibiting the malignant transformation of oral potentially malignant disorders (OPMDs) and for improving post-treatment quality of life by promoting tissue regeneration for postoperative tissue defects. Figure created using BioRender. Liang, J. (2025), https://BioRender.com/msdengq, accessed on 7 April 2025.

This review comprehensively explores four dimensions of PTT in oral oncology: (1) strategies to enhance PTAs’ performance; (2) synergistic PTT-based combinatorial therapies; (3) multifunctional PTT systems integrating tumor ablation and tissue repair; and (4) PTT applications in OPMDs management (Figure 1). We highlight cutting-edge nano platform designs, targeted delivery mechanisms, and clinical translation challenges (such as long-term biosafety and temperature control) (Table 1). By bridging preclinical innovation and clinical needs, this work aims to catalyze the development of precision-engineered, multifunctional PTT strategies to improve therapeutic outcomes and enhance the overall patient survival rate in oral cancer.

## 2. Strategies to Enhance PTT Therapeutic Effectiveness

The efficacy of PTT in treating oral cancer hinges on addressing three critical challenges: enhancing the PCE of PTAs, optimizing tumor-specific accumulation, and determining the optimal laser irradiation timing. Augmenting PCE of PTAs reduces the required agent concentration for tumor ablation, thereby minimizing long-term biotoxicity while concurrently lowering laser power and exposure duration to improve therapeutic safety and efficiency [121]. Enhancing tumor-targeted accumulation of PTAs mitigates off-target thermal damage to healthy tissues, a cornerstone strategy for amplifying PTT efficacy in oral oncology [122,123]. Furthermore, the dynamic biodistribution of PTAs necessitates precise temporal control, as irradiation during peak intratumoral accumulation maximizes ablation efficacy and minimizes collateral effects [124]. Real-time imaging-guided PTT, leveraging techniques such as photoacoustic tomography or fluorescence tracking, could synchronize laser activation with PTAs’ pharmacokinetic peaks, enabling spatiotemporally precise therapy [125].

### 2.1. Selection of Appropriate PTAs

PTT hinges on PTAs to convert light energy into thermal energy, making PTAs the cornerstone of PTT efficacy. For optimal performance, PTAs should demonstrate a high PCE. Nanoparticles (NPs), owing to their strong NIR absorption capabilities, are widely utilized due to their superior PCE [126,127]. Nanomaterials employed in PTT for oral cancer can be categorized into four classes: noble metal nanomaterials, metal compounds, carbon-based nanomaterials, and organic nanoparticles [53]. Upon electromagnetic excitation, metallic nanoparticles exhibit localized surface plasmon resonance (LSPR), leading to intense electromagnetic energy absorption and subsequent heat generation through electron excitation and relaxation—a mechanism known as plasmonic photothermal therapy (PPTT), which demonstrates superior light absorption and thermal conversion efficiency compared to conventional PTT [128,129]. Coupled with inherent biocompatibility, these properties have driven the widespread adoption of metallic nanoparticles [130].

Among noble metal-based PTAs, gold nanomaterials (AuNPs) stand out as the most extensively studied due to their exceptional photothermal performance, tunable size/morphology, and low toxicity [65]. The heat generation capacity of AuNPs under NIR irradiation depends on their absorption cross-section and efficiency, which are governed by particle geometry and dimensions. Gold nanorods (AuNRs), in particular, exhibit optimal NIR absorption cross-sections and unparalleled PCE among plasmonic AuNPs [64]. Their photothermal performance is further modulated by factors such as absorption-to-extinction ratio, near-field electric field intensity, and decay length of the electric field beyond the nanoparticle surface [131]. Size-dependent studies reveal that 28 × 8 nm AuNRs outperform 17 × 5 nm or 38 × 11 nm counterparts in photothermal ablation efficiency, as demonstrated by Mackey et al. [59], establishing them as a gold standard for cancer cell eradication.

### 2.2. Accumulation of PTAs in Tumor Tissue

**Figure 2:**
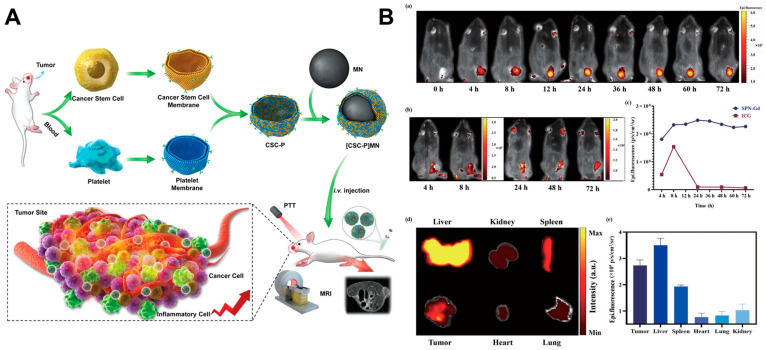
Strategies to enhance PTT therapeutic effectiveness. (**A**) Schematic illustration of [CSC-P]MNs and their application in cancer theranostics. Reproduced with permission [70]. Copyright 2019, John Wiley and Sons Group (Hoboken, NJ, USA). (**B**) (**a**) Fluorescence images at different time points following injection of SPN-Gd, (**b**) fluorescence images at different time points following indocyanine green injection, (**c**) comparison of fluorescence signal intensity between SPN-Gd and indocyanine green at different time points, (**d**,**e**) fluorescence signal intensity of main organs and tumors 72 h following injection of SPN-Gd. Reproduced with permission [74]. Copyright 2022, Springer Nature Group (Berlin, Germany).

The insufficient intratumoral accumulation of PTAs necessitates higher dosages, prolonged laser exposure, or increased laser power, raising risks of off-target tissue damage. Thus, targeted delivery and retention of PTAs within tumors are critical for safe and efficient phototherapy. A key advantage of nanomaterial-based cancer therapy lies in exploiting the tumor’s unique vascular leakage [132]. Rapid angiogenesis within tumors leads to aberrant vasculature and compromised lymphatic drainage, collectively giving rise to the enhanced permeability and retention (EPR) effect—an inherent “passive targeting” mechanism within the tumor microenvironment [133]. However, despite leveraging EPR-driven passive targeting, PTAs accumulation in tumors remains suboptimal, necessitating more efficient and active targeting strategies to enhance tumor-specific cellular uptake and minimize nonspecific biodistribution [61]. Active targeting exploits overexpressed cell surface markers (such as receptors) to selectively direct PTAs to cancer cells [134,135]. Nanoparticles possess a high surface-area-to-volume ratio, enabling dense surface functionalization with various targeting moieties, including antibodies, small molecules, peptides such as anti-epidermal growth factor receptor (anti-EGFR) [64], HN-1 peptide [54], SERPINH1 antibody [135], Arg–Gly–Asp (RGD) peptide [136]. Compared with PTT alone, active targeting enables malignant cell ablation at lower temperatures, requiring only half the energy needed to disrupt normal cells at equivalent nanorod-bioconjugate concentrations, thereby enhancing therapeutic specificity and reducing side effects [64]. Biomimetic nanoparticles coated with cell membranes derived from stem cells [70], cancer cell membranes [55,137], or platelets [63] have recently gained attention due to their superior biocompatibility and homologous targeting properties. For instance, Bu et al. [70] demonstrated that iron oxide magnetic nanoparticles coated with a platelet-cancer stem cell hybrid membrane ([CSC-P]MNs) resulted in greater tumor accumulation, less uptake by the spleen and liver, and diminished metabolic activity, thus enhancing PTT therapeutic efficacy (Figure 2A). These coatings prolong circulation time and enhance tumor accumulation through immune evasion and tissue-specific homing.

Drug delivery systems (DDS) co-loaded with PTAs further optimize therapeutic outcomes. Nanoparticle-based DDS improves pharmacokinetics by enhancing drug stability, extending half-life, and preventing premature release [138]. Stimuli-responsive DDS, activated by tumor microenvironment cues (e.g., pH [95,96]) or external triggers (e.g., light [77,99]), enable spatiotemporally controlled drug release [139]. For instance, pH-responsive charge-reversal nanodrug systems initially maintain a negative surface charge in circulation to minimize clearance [72,98]. Upon reaching acidic tumor regions, dimethylmaleamide cleavage converts the surface charge to positive, enhancing tumor penetration, cellular uptake, and localized drug release [72]. Coupled with intrinsic photothermal properties, such systems achieve synergistic tumor ablation and chemotherapy.

While intravenous administration dominates PTAs delivery, the superficial location of oral cancers enables alternative localized strategies [140,141]. Injectable hydrogels, administered via peritumoral or intratumoral routes, offer precise PTAs deposition. For example, E72-chitosan hydrogels injected around tongue tumors degrade within 24 h, ensuring prolonged local retention of inorganic NPs [68]. Single-session PTT with this approach achieves complete tumor eradication without functional compromise or systemic toxicity. Intratumoral injection further concentrates PTAs within lesions, maximizing hyperthermia efficacy. Post-PTT, residual PTAs are expelled alongside necrotic tissue, eliminating systemic absorption risks [50].

### 2.3. Navigation from Imaging

Systemic administration of PTAs in many studies results in their circulation through the vasculature and subsequent accumulation in tumors via the EPR effect. The pharmacokinetic properties of PTAs exhibit significant variability based on their physicochemical characteristics [124]. Optimal therapeutic outcomes are typically achieved when PTT is initiated at peak intratumoral PTAs accumulation. To address this, researchers have integrated imaging-guided strategies to monitor PTAs biodistribution in real-time, enabling laser irradiation at maximal tumor retention and dynamic adjustment of dosing regimens [84,85]. This approach minimizes off-target toxicity while maximizing therapeutic precision. Furthermore, tumor-targeted imaging facilitates diagnostic applications, such as preoperative tumor localization and postoperative detection of residual malignant cells [75,76]. For instance, nitrogen-rich mesoporous carbon nanospheres were synthesized using Triton-X as a structural template and pyrrole/aniline precursors. These luminescent hollow nanospheres combine PTT with fluorescence (FL) imaging capabilities, exhibiting a fluorescence quantum yield of 14.6%. The nanospheres induced potent thermal ablation of tumor cells, while their FL properties enabled real-time tracking of therapeutic responses [73]. Moreover, multimodal bioimaging, including different imaging platforms simultaneously, could improve the accuracy of imaging guidance and adjuvant therapy since the advantages and disadvantages of the different imaging modes complemented each other [75]. Pan et al. [74] have developed gadolinium-containing semiconductor polymer nanoparticles (SPN-Gd) that function as both an FL signal source and magnetic resonance imaging (MRI) contrast agents, thus offering a simplified nanotheranostic platform for efficient MRI/FL dual-modal imaging-guided PTT. Following intravenous administration of nanoparticles, FL signals were observed at the tumor site and progressively increased, peaking at 24 h and remaining elevated from 24 to 72 h. The maximum fluorescence signal of SPN-Gd detected at 24 h further implied that PTT should be performed 24 h following the injection of SPN-Gd. Furthermore, the biodistribution of SPN-Gd was assessed by FL imaging at 72 h post-injection, revealing the liver as having the highest fluorescence signal, followed by the tumor, spleen, and other major organs. (Figure 2B). Beyond MRI and FL, many imaging platforms—including computed tomography (CT) [54,75], light scattering imaging [64], and photoacoustic (PA) imaging [83]—have been synergized with PTAs to enhance diagnostic-therapeutic integration. The simultaneous use of diverse imaging platforms in multimodal bioimaging can improve the precision of imaging guidance and adjuvant therapy, as the advantages and disadvantages of different imaging modes are mutually compensating [75]. Noninvasive, real-time monitoring of treatment progression and feedback-driven adjustments are pivotal for translating preclinical successes into clinical practice. By bridging imaging precision with therapeutic spatiotemporal control, these advancements herald a new era of personalized oncology, where PTT is tailored to individual tumor dynamics and patient-specific pharmacokinetics.

## 3. Synergistic PTT-Based Therapies

The inherent diversity, complexity, and heterogeneity of tumors often render monotherapy insufficient for complete eradication [142]. To address this, combinatorial therapies can be strategically designed to exploit tumor and microenvironmental heterogeneity, integrating multiple modalities to achieve synergistic antitumor effects [85]. Such approaches enable comprehensive therapeutic outcomes while reducing individual treatment dosages without compromising efficacy—or even enhancing it [95,96]. PTT-based synergistic systems are particularly advantageous, as localized hyperthermia acts as a potent sensitizer by altering chemotherapeutic pharmacokinetics, modulating cell membrane permeability/stability, enhancing tissue oxygenation, and disrupting DNA repair mechanisms critical for tumor recovery [114,115,116,117]. These thermal-driven modifications amplify the cytotoxic effects of chemotherapy, immunotherapy, or radiotherapy, overcoming resistance mechanisms inherent to heterogeneous tumor subpopulations.

### 3.1. PTT Combined with PDT

**Figure 3:**
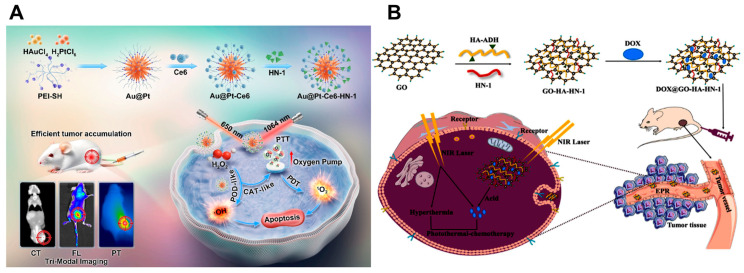
Synergistic PTT based therapies. (**A**) Schematic illustration of the key preparation steps for the Au@Pt-Ce6-HN-1 nanoplatform and its synergistic PTT/PDT antitumor mechanism. Reproduced with permission [85]. Copyright 2024, Elsevier Group (Amsterdam, The Netherlands). (**B**) Schematic illustration of the synthesis of dual-targeting nanoparticles DOX@GO-HA-HN-1 and their application in chemotherapy-synergized PTT for oral cancer. Reproduced with permission [94]. Copyright 2024, Elsevier Group (Amsterdam, The Netherlands).

PDT eliminates tumors by converting oxygen into reactive oxygen species (ROS) via photosensitizers (PS) under light irradiation. However, the hypoxic TME severely limits PDT efficacy, hindering its clinical application [143]. In contrast, PTT operates independently of oxygen availability and can synergistically enhance PDT through mild hyperthermia. Initial mild heating increases tumor blood flow and endothelial gaps, transiently improving oxygenation to enable effective PDT while enhancing nanocarrier delivery to tumors. Subsequent PDT following PTT thus achieves superior therapeutic outcomes [144]. ROS generated during PDT also sensitizes cancer cells to PTT-induced hyperthermia, creating a bidirectional therapeutic amplification loop [79]. Although some studies employ dual laser sources to activate PTT and PDT independently, this complicates clinical workflows [78]. Most research focuses on single-laser irradiation, which synchronizes PTT and PDT activation, minimizes treatment latency, and enhances synergy [139,145]. Photothermal nanoparticles can serve as PS carriers by matching their LSPR wavelength with the PS excitation spectrum, enabling single-laser excitation. In such systems, the intense near-field distribution generated by LSPR around plasmonic NPs (such as AuNPs) enhances PS excitation efficiency [80]. For example, Li et al. [85] fabricated a multifunctional Au@Pt-Ce6-HN-1 nanoplatform. They synthesized sea urchin-like Au@Pt nanozymes using a simple seed-mediated growth method and subsequently conjugated chlorin e6 (Ce6) and the HN-1 peptide (TSPLNIHNGQKL) to the Au@Pt surface via amide reactions. This platform exhibits exceptional photothermal and photodynamic performance, enabling synergistic PTT/PDT for enhanced tumor therapy (Figure 3A). Another nanoplatform that integrates indocyanine green (ICG) with hyaluronic acid-coated single-walled carbon nanotubes (SWCNT-HANPs) was developed to co-activate PTT and PDT. ICG’s thermal stability ensures simultaneous photothermal and photodynamic effects under NIR irradiation [83]. These dual-modal systems exemplify the potential of rational nanomaterial design to overcome the intrinsic limitations of standalone therapies, offering a blueprint for next-generation combinatorial strategies.

### 3.2. PTT Combined with Chemotherapy

Chemotherapy employs cytotoxic agents to eliminate tumor cells, suppress proliferation, and eradicate residual malignancies [146]. However, systemic toxicity—such as myelosuppression, gastrointestinal distress, and alopecia—limits its clinical utility [147]. Thus, achieving on-demand controlled drug release is critical, requiring nanocarriers to balance premature drug leakage in circulation with rapid payload discharge at tumor sites. PTT has emerged as a potent external stimulus for tumor-specific and sequential drug release. Mechanisms include photothermal-mediated carrier disruption (e.g., thermal lysis of liposomes) or cleavage of thermolabile chemical bonds (e.g., Au-S bonds), with laser intensity modulating release kinetics [53]. Moreover, PTT remodels the TME by enhancing vascular permeability and interstitial fluid pressure, thereby promoting nanocarrier penetration and intratumoral accumulation [148]. The synergistic integration of PTT and chemotherapy amplifies therapeutic efficacy through dual pathways: (1) localized hyperthermia directly sensitizes cancer cells to chemotherapeutics, and (2) photothermally enhanced drug delivery enables the eradication of metastatic lesions beyond the irradiation field [55,149]. For example, Li et al. [94] have developed a novel dual-targeting nano-delivery system for the combined effects of PTT and chemotherapy through the co-modification of carboxylated graphene oxide (GO) with hyaluronic acid (HA) and HN-1 peptide followed by the encapsulation of the anticancer drug doxorubicin (DOX) (Figure 3B). Other exemplary systems include gold nanoflowers with DOX [99], gold nanorods conjugated to vincristine (VCR) [89], Prussian blue nanoparticles loaded with DOX [100], and ultrasmall gold nanoparticles complexed with cisplatin [91]. These platforms demonstrate superior antitumor outcomes by harmonizing photothermal ablation with spatiotemporally controlled chemotherapy.

### 3.3. PTT Combined with Radiotherapy

Radiotherapy (RT) employs high-energy ionizing radiation to eradicate cancer cells, commonly administered before or after surgical tumor resection to eliminate residual malignancies and prevent recurrence [150]. However, RT predominantly targets tumor cells in the G2/M phase, exhibiting limited efficacy against S-phase cells [151]. Its therapeutic impact is further constrained by tumor hypoxia, a key contributor to post-RT relapse and metastasis [152,153]. Additionally, RT inflicts severe collateral damage on normal tissues exposed to ionizing radiation [154]. PTT synergistically enhances RT efficacy by improving tumor oxygenation through hyperthermia-induced blood flow augmentation. This combinatorial approach leverages the distinct anticancer mechanisms of RT and PTT to overcome therapeutic resistance. AuNPs, owing to their megavolt or kilovolt X-ray absorption properties, have emerged as novel radiosensitizers in preclinical studies [155]. Radiation enhancement by these agents is mediated by both physical dose deposition (through photoelectric and Compton interactions) and biological mechanisms such as amplifying oxidative stress, disrupting the cell cycle, and inhibiting DNA repair [156]. For instance, Neshastehriz et al. [103] synthesized folate-conjugated AuNPs (F-AuNPs) that selectively accumulate in tumors via folate receptor-mediated targeting. Under 532 nm laser irradiation, F-AuNPs induced significant thermal ablation due to their localized photothermal conversion. Simultaneously, their high Z-value enhanced radiosensitization during X-ray exposure. In cancer cells treated with F-AuNPs, dual irradiation (laser + X-ray) reduced cell viability, far exceeding the effects of PTT or RT alone. This dramatic synergy underscores the potential of AuNP-mediated RT-PTT integration to achieve precision tumor eradication while sparing healthy tissues.

### 3.4. PTT Combined with Gene Therapy

Gene therapy can induce apoptosis in malignant cells and downregulate heat shock protein (HSP) expression to mitigate cellular protection against photothermal-induced hyperthermia or upregulate cytotoxic immune cytokines [157]. Researchers have employed gene silencing to counteract thermoresistance caused by the heat shock response (HSR) and synergized this strategy with PTT for treating OSCC [158]. The HSR, a defense mechanism triggered by thermal or other stressors, involves adaptive gene expression changes that enhance cancer cell resistance to therapeutic heat, thereby diminishing PTT efficacy [159]. To address this, small interfering RNA (siRNA) has been utilized to inhibit HSR signaling, which is mediated by HSPs such as HSP70 [57] and BAG3 [104,160]. For instance, Wang et al. [104] developed a gold nanorod-siRNA (GNRs-siRNA) platform with gene-silencing capabilities. This nanoplatform sensitizes cancer cells to PTT using moderate laser irradiation by downregulating BAG3 expression and promoting apoptosis, effectively overcoming thermoresistance. GNRs-siRNA-mediated PTT eliminates therapeutic resistance via targeted gene silencing, amplifies photothermal efficacy, and maximizes tumor eradication while minimizing off-target tissue damage, demonstrating significant potential for clinical translation in precision oncology.

### 3.5. PTT Combined with Immunotherapy

Immunotherapy harnesses the intrinsic capacity of the cellular and humoral immune systems to target and eliminate tumor cells directly, offering high therapeutic specificity and safety [161]. Current strategies to stimulate antitumor immunity include checkpoint blockade therapy, adoptive T cell transfer, and cancer vaccines [162,163]. By enhancing the maturation of antigen-presenting cells (APCs) and cytotoxic T lymphocytes (CTLs) and suppressing immunosuppressive mechanisms within tumors, immunoadjuvants or checkpoint inhibitors amplify immune activation [162]. The integration of PTT with immunotherapy creates synergistic anticancer effects through dual mechanisms: (1) PTT-induced immunogenic cell death releases tumor-specific antigens in situ, priming adaptive antitumor immune responses and constraining tumor growth by eliciting immunogenic cell death, (2) host immune activation eradicates disseminated or metastatic lesions beyond the laser-irradiated tumor field, preventing recurrence [53]. For example, Bu et al. [105] synthesized matrix metalloproteinase (MMP)-degradable gelatin nanoparticles (GNPs) co-loaded with ICG and a signal transducer activator of transcription 3 (STAT3) inhibitor (NSC74859) to construct Gel-N-ICG NPs. The released NSC inhibits immune checkpoint proteins and induces robust antitumor immunity, significantly suppressing tumor growth. Under NIR irradiation, Gel-N-ICG NPs simultaneously mediate photothermal ablation and immune checkpoint blockade, achieving combinatorial tumor eradication and systemic immune memory. High cholesterol in the TME may affect T cells’ function, leading to the depletion of CD8^+^ T cells’ immune function and other functional abnormalities. Song et al. [115] developed a cholesterol-regulating nanoplatform comprised of simvastatin (SIM) encapsulated within chlorin e6-polyethylene glycol (Ce6-PEG). Simvastatin acts by inhibiting hydroxy-3-methylglutaryl-CoA reductase (HMGCR), thereby lowering cholesterol levels in tumor tissues and reversing the immunosuppressive effects of tumor-infiltrating T cells. It unlocked new paradigms to provide a TME-regulated-based scaffold for practical cancer applications.

## 4. PTT-Based Multifunctional Therapeutic Platform

Currently, surgical resection remains the primary treatment for oral cancer. However, residual tumor cells at surgical margins frequently lead to recurrence [164]. Occurrence of positive surgical margins, indicating residual malignant cells, is reported in 20–50% of OSCC cases—a frequency 1.7 times higher compared to other head and neck locations—which continues to hinder the attainment of favorable postoperative outcomes [165,166,167]. Adjuvant therapies, such as radiotherapy and chemotherapy, are often required to eliminate residual tumor cells but are associated with severe side effects, including osteoradionecrosis and rampant caries, which profoundly impair patients’ quality of life [168,169]. Moreover, extensive bone and soft tissue defects caused by surgery compromise aesthetic and functional outcomes (such as mastication, speech), imposing significant psychological and physiological burdens [170,171]. Autologous tissue transplantation, the clinical “gold standard” for defect repair, faces limitations such as donor site morbidity, postsurgical hair growth in flaps, and technical complexity [172,173,174,175]. Thus, eliminating residual tumor cells while promoting tissue regeneration is critical in postoperative care. Demonstrated in the treatment of infectious bone defects, NIR-mediated mild hyperthermia enhances cell proliferation, differentiation, and local blood flow, enabling PTT to function as a postoperative adjunct for eradicating residual tumor cells and stimulating tissue repair [176,177,178,179]. In addition, surgical exposure of the tumor region adds to its advantages. However, standalone PTT exhibits limited regenerative capacity [180]. Recent advances in tissue engineering have spurred interest in integrating PTT with biomimetic scaffolds to construct multifunctional systems that synergize antitumor efficacy and tissue regeneration [181,182,183].

**Figure 4:**
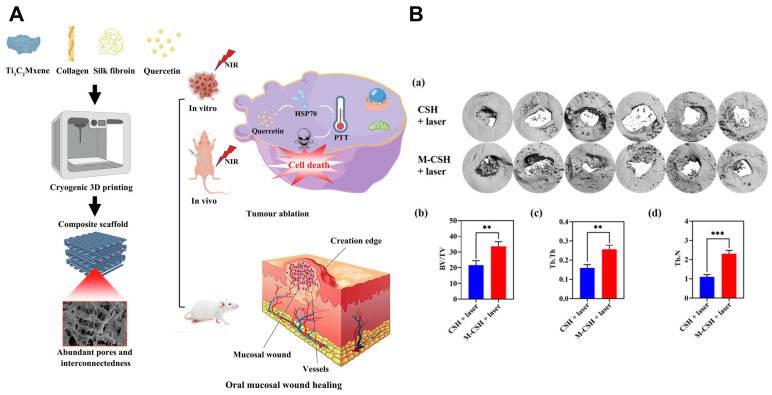
PTT based multifunctional therapeutic platform. (**A**) Illustration outlining the preparation scheme of M-CSQ scaffolds and their role in combinational PTT against oral cancer cells and oral mucosal wound repair. Reproduced with permission [57]. Copyright 2023, Elsevier Group (Amsterdam, The Netherlands). (**B**) Osteogenesis performance of CSH and M-CSH scaffolds. (**a**) Micro-CT images of mandibular defect areas at 8 weeks after surgery, the value of (**b**) BV/TV, (**c**) Tb. Th and (**d**) Tb. N in new bone tissue. ** *p* < 0.01, *** *p* < 0.001. Reproduced under the terms of the Creative Commons CC BY license [56]. Copyright 2021, The Author(s). Published by Oxford University Press (Oxford, UK).

Bioinspired scaffolds are emerging as promising platforms for oral wound repair [184]. These scaffolds provide structural guidance for cell migration and proliferation, facilitating tissue formation. Medical-grade silk fibroin (SF), renowned for its biocompatibility, elasticity, and tunable biodegradability, is widely employed in scaffold design [185]. Collagen (COL) further enhances scaffold performance by promoting platelet adhesion, fibroblast proliferation, and angiogenesis during early wound healing [186]. Three-dimensional printing enables the fabrication of personalized scaffolds with interconnected macroporous architectures tailored to irregular defects. Accordingly, many research efforts have focused on developing 3D-printed biomimetic scaffolds composed of SF/COL [57]. For instance, PTT and tissue engineering scaffold were rationally integrated to construct a Ti_3_C_2_ MXene, COL, SF, and quercetin composite scaffold (M-CSQ), a 3D printed biomaterial that simultaneously kills OSCC cells and promotes the regeneration of the mucosal defects. The M-CSQ scaffolds were prepared using cryogenic 3D printing and freeze-drying techniques, with sufficient pores that allow abundant cell migration and provide a proliferation space. The results suggest the achievement of concurrent residual OSCC cell elimination and oral mucosal wound repair (Figure 4A) [57]. Short-term, controlled laser exposure during PTT minimally impacts long-term mucosal regeneration, underscoring its clinical feasibility [58]. In advanced oral cancers invading maxillofacial bone, surgical ablation often results in critical-sized defects requiring reconstruction. Nanohydroxyapatite (nHA)-incorporated scaffolds, mimicking bone’s inorganic composition, offer osteoconductivity and mechanical robustness [187,188]. To simultaneously eradicate residual tumor cells and regenerate bone, a 3D-printed collagen-silk-nHA (CSH) scaffold was functionalized with MXene nanosheets (M-CSH) [56]. Both the CSH and M-CSH scaffolds were shown to support the generation of new bone, with the M-CSH scaffolds exhibiting a superior ability to form bone compared to the CSH scaffolds, even with short-term PTT (Figure 4B). This indicates that short-term PTT does not adversely impact long-term bone repair outcomes. Although localized heat from NIR irradiation may transiently affect adjacent osteocytes, bone marrow mesenchymal stem cells (BMSCs) recruited during the inflammatory phase migrate into scaffold pores, proliferate, and differentiate, ultimately restoring osseous architecture. Notably, PTT-induced inflammation mirrors the initial bone healing phase, enhancing BMSC homing to defect sites [189].

## 5. PTT for OPMDs Management

Oral potentially malignant disorders (OPMDs)—including conditions such as leukoplakia, erythroplakia, oral lichen planus, proliferative verrucous leukoplakia, and oral submucous fibrosis—significantly increase the risk of OSCC development [14]. Current clinical management strategies for OPMDs encompass surgical resection, cryotherapy, laser ablation, PDT, and pharmacotherapy [190]. However, anatomical and functional constraints in the orofacial region often limit the applicability of invasive modalities like surgery or laser therapy [119]. With its minimally invasive nature, high selectivity, repeatability, and minimal scarring, PTT has emerged as an indispensable approach for treating OPMDs, particularly in functionally sensitive areas.

**Figure 5:**
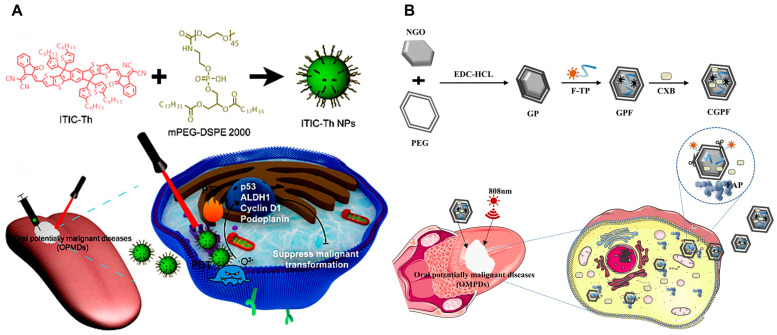
PTT for oral potentially malignant disorders (OPMDs) management. (**A**) Schematic illustration of the synthesis of ITIC-Th NPs and their synergistic PDT/PTT mechanism. Reproduced under the terms of the Creative Commons CC BY license [118]. Copyright 2022, The Author(s). Published by Springer Nature Group (Berlin, Germany). (**B**) Illustration depicting the synthesis of the nano-drug delivery system and describing the combined PTT approach. Reproduced with permission [119]. Copyright 2023, Springer Nature Group (Berlin, Germany).

Currently, PTT applications in OPMDs remain limited, primarily focusing on oral leukoplakia (OLK)—the most prevalent OPMD, defined as a non-removable, non-scrapable white plaque with malignant potential [29,191]. OLK exhibits a 3.5% prevalence rate, with 4–13% progressing to malignancy [192,193]. Preventing OPMD-to-OSCC transformation is critical. For instance, ITIC-Th, a dual-modal PDT/PTT agent, synergistically induces cell death by downregulating tumor-associated biomarkers (such as ALDH1, p53, cyclin D1, PDCD5, and PDPN) in vitro, effectively blocking malignant transformation and promoting OLK lesion regression. Concurrently, PTT enhances local blood flow to accelerate tissue healing (Figure 5A) [115]. Nevertheless, nonspecific thermal diffusion during PTT risks collateral damage to adjacent healthy tissues. To address this, fibroblast activation protein (FAP)—overexpressed in epithelial tumors and inflammatory lesions but absent in normal adult tissues—has been leveraged to design FAP-targeted nanodrug delivery systems (Figure 5B) [119]. Soluble microneedles have also been used as in situ transdermal delivery systems to decrease the risk of damage to surrounding normal tissue [120]. These systems achieve localized therapy by selectively accumulating in lesions, maximizing therapeutic precision while minimizing off-target effects.

## 6. Summary and Outlook

PTT has emerged as a promising anticancer modality owing to its non-invasive nature, precise spatiotemporal control, and resistance-free mechanism, offering transformative potential for both the prevention and treatment of oral cancer. Advances in nanotechnology have driven the rational design of PTAs through material selection, morphological optimization, and size modulation to maximize PCE and therapeutic outcomes. Concurrently, strategies to enhance the tumor-specific accumulation of PTAs—via active targeting (e.g., ligand-receptor interactions) and image-guided timing of laser irradiation—have significantly improved treatment precision. The integration of PTT with multimodal therapies, including conventional approaches (surgery, radiotherapy, chemotherapy) and emerging modalities (gene therapy, PDT, immunotherapy), addresses the limitations of monotherapy by leveraging synergistic effects. For instance, PTT-induced hyperthermia sensitizes tumors to chemotherapy while mitigating systemic toxicity, and combinational PTT-immunotherapy eradicates metastatic lesions via systemic immune activation. Notably, the fusion of PTT with tissue-engineered scaffolds represents a groundbreaking approach, enabling simultaneous tumor ablation and functional tissue regeneration—a critical advancement for reconstructing maxillofacial defects post-resection. While this review categorizes PTT strategies individually, real-world applications often combine multiple approaches (e.g., targeted PTAs + image guidance + scaffold integration) to amplify efficacy [96,98]. Furthermore, strategies developed for oral cancer—such as optimized PTA design and combinatorial regimens—hold untapped potential for OPMDs, given the shared therapeutic vulnerabilities between OPMDs and malignant cells.

Despite the preclinical success, clinical translation faces challenges: (1) Limited ablation efficacy in bulky tumors, (2) Safety concerns regarding systemic nanomaterial administration, (3) Reliance on cell line-derived xenograft models, which poorly replicate human tumor heterogeneity and microenvironmental complexity. Future efforts should prioritize (1) Developing PTAs with high extinction coefficients and optimal absorption/scattering ratios for NIR-II window (1000–1700 nm) applications, which offer deeper tissue penetration and higher permissible irradiation doses. (2) Exploring localized delivery systems (e.g., hydrogels, nanocapsules, hyaluronic acid-based carriers) to minimize systemic toxicity. (3) Adopting patient-derived xenograft (PDX) models to predict clinical outcomes better. (4) Investigating mechanisms underlying mild hyperthermia-driven tissue regeneration, currently attributed to exogenous factor release but lacking mechanistic depth. (5) Accelerating clinical trials to validate preclinical findings, as current human studies remain scarce. In conclusion, PTT stands at the frontier of oral oncology, with transformative potential to bridge tumor eradication and functional restoration. Through interdisciplinary innovation and rigorous clinical validation, PTT-based paradigms may soon redefine standards of care for oral cancer and OPMDs.

## Figures and Tables

**Table 1:** Summaries of photothermal therapy (PTT) for oral cancer and oral potentially malignant disorders (OPMDs).

Disease Type	Therapy Mode	Photothermal Agents	Functionalization	Exposure Conditions	Tumor Type	Therapeutic Highlights	Ref.
Oral Cancer	PTT	AuNRs	PEG conjugated	808 nm, 5.8 W cm^−2^	HSC 3 cells	28 × 8 nm AuNRs are a more effective photothermal contrast agent for PTT of OSCC	[59]
			Thiolated PEG conjugated	808 nm, 0.9–1.9 W cm^−2^	HSC 3 cells, CDX mouse model	Preferential tumor accumulation of Pegylated AuNRs indicates the selectivity and specificity of PTT	[60]
			Folate conjugated	755 nm, 40 J cm^−2^	KB cells	Folic acid for active tumor targeting	[61]
			Cysteine-functionalized alginate and cyclic peptide, c(RGDfK)KKK modified	808 nm, 2 W cm^−2^	SAS 3 cells, CDX mouse models	Replacing cetyltrimethylammonium bromide (CTAB) with alginate improves the biocompatibility of AuNRs	[62]
			PLTs loaded	808 nm, 2 W cm^−2^	CAL 27 cells, CDX mouse model, HNSCC-bearing *Tgfbr1/Pten* 2cKO mouse model	PTT enhances tumor targeting of PLT-AuNRs, which in turn improves PTT effects via a feedback mechanism, demonstrating the benefit of PLT-PTT in cancer therapy	[63]
			Anti-EGFR antibodies conjugated	800 nm, 10–20 W cm^−2^	HOC 313 cells, HSC 3 cells	Integrated molecular imaging with photothermal cancer therapy	[64]
		AuNPs	—	532 nm, 0.3 W cm^−2^	DMBA-induced HBP carcinoma	Plasmonic photothermal therapy on induced HBP carcinoma	[65]
			Anti-EGFR antibodies conjugated	514 nm, 13–64 W cm^−2^	HSC 313 cells, HOC 3 cells	Selective PTT for epithelial carcinoma	[66]
		CuS NPs	BSA-templated synthesis of BSA@CuS nanoparticles with subsequent PEGylation	1064 nm, 0.5 W cm^−2^	CAL 27 cells, SCC 9 cells, CDX mouse model	Modified with PEG to increase biocompatibility	[67]
		Ag_3_AuS_2_ NPs	Ag_3_AuS_2_ NPs complexed with genetically engineered anionic protein and chitosan	808 nm, 1 W cm^−2^	CAL 27 cells, CDX mouse model	Features tongue tumor inhibition and complication prevention	[68]
		Gold-silica nanoshells	Anti-HER2 nanobodies conjugated	820 nm, 4 W cm^−2^	KB cells	First in vitro investigation of PTT efficacy using anti-HER2 nanobody-conjugated nanoshells in an OSCC model	[69]
		Fe_3_O_4_ NPs	Platelet-cancer stem cell hybrid membrane coated	808 nm, 5 W cm^−2^	CAL 27 cells, CDX mouse model, HNSCC-bearing *Tgfbr1/Pten* 2cKO mouse model	First presentation of a platelet–cancer stem cell hybrid membrane-coated iron oxide magnetic nanoparticle for enhanced PTT of HNSCC	[70]
		Au@C NPs	Membrane of patient-derived cells coated	808 nm, 1 W cm^−2^	CAL 27 cells, SCC 7 cells, HN 6 cells, CDX mouse model, orthotopic tongue tumor mouse model, primary and distant tumor mouse models, PDX mouse model	The homologous cancer cell membrane provided the nanoplatforms with optimal targeting properties for maximum therapeutic efficiency	[71]
		BP NSs	PDA and polyacrylamide hydrochloride-dimethylmaleic acid (PAH-DMMA) charge reversal system modified	808 nm, 1.5 W cm^−2^	CAL 27 cells, SAS cells, CDX mouse model	Nanoplatforms exhibit suitable size for intravenous delivery, enrichment in tumor sites, enhanced tumor cell uptake, excellent photothermal properties, and effective oral cancer cell killing	[72]
		Carbon dots	Triton-X-directed synthesis of N-rich mesoporous carbon nanospheres from pyrrole and aniline	980 nm, 1.4 W cm^−2^	FaDu cells	Exhibiting integrated PTT and FL functionalities	[73]
		Semiconductor polymer (PCPDTBT)	Incorporated gadolinium-grafted triblock amphiphilic copolymer (F127-DTPA-Gd)	808 nm, 1 W cm^−2^	SCC 7 cells, CDX mouse model	Two-component nanotheranostic platform enabling efficient MRI and FL-guided PTT	[74]
		GO	Amino-modified	808 nm, 2 W cm^−2^	HSC 3 cells, CDX mouse model	Graphene-based nanomaterials as direct nano-PTAs for anticancer PTT	[50]
		Gold nanodots	Peptide HN-1 modified	808 nm, 2 W cm^−2^	SCC 9 cells, CAL 27 cells, CRL 1623 cells, CDX mouse model	Versatile nanosystem for targeted drug delivery and diagnostic imaging	[54]
		Au/Mn nanodots	—	1064 nm, 2 W cm^−2^	SCC 9 cells, CAL 27 cells, CDX mouse model	Features multimodal bioimaging, including concurrent CT and MRI, and bright near-infrared FL for navigation	[75]
		ICG	Functionalized with cypate fluorophore and two cyclic-(arginine-glycine-aspartic acid) (cRGD) peptides	808 nm, 1 W cm^−2^	CAL 27 cells, CDX mouse model	ICG-derived NIR fluorescent probes designed and synthesized for accurate diagnosis and treatment	[76]
		Aggregation-induced emission luminogens (AIEgen)	NMB@NPs constructed from carbon radical monomer, ethyl 2,6-diisocyanatohexanoate, PEG molecules, and an AIEgen	808 nm, 1 W cm^−2^	CAL 27 cells, PDX mouse model	FL-guided thermodynamic therapy and PTT	[77]
		MXene NSs	Incorporated into a scaffold created from collagen, silk, and hydroxyapatite	808 nm, 1 W cm^−2^	CAL 27 cells, bone defect rabbit model	Simultaneously kills OSCC cells and promotes bone tissue regeneration	[56]
		Ti_3_C_2_ Mxene	Scaffold constructed from Ti_3_C_2_ MXene, collagen, and silk fibroin	808 nm, 1 W cm^−2^	SCC 25 cells, CAL 27 cells, CDX mouse model	Exhibits simultaneous OSCC cell cytotoxicity and mucosal defect regeneration	[57]
		ICG	Scaffold fabricated with collagen/silk fibroin and ICG	808 nm, 1 W cm^−2^	SCC 25 cells, CDX mouse model	Facilitated the attachment and proliferation of rat buccal mucosa fibroblasts and enhanced the repair of buccal mucosal wounds	[58]
	PTT, PDT	AuNRs	Rose bengal molecules conjugated	810 nm, 17.86 W cm^−2^, 532 nm, 1.76 W cm^−2^	CAL 27 cells, DMBA-induced HBP carcinoma	Combined PDT/PTT capabilities against oral cancer	[78]
		Organic compound (C3)	ICG and C3 encapsulated within PEG-PCL	808 nm, 0.5 W cm^−2^	HSC cells, CDX mouse model	FL-guided PTT/PDT against OSCC	[45]
		Au nanoflower	Two layers of silica shell ICG added	808 nm, 10 W cm^−2^	Cal 27 cells, CDX mouse model	Tumor growth inhibition through synchronous PTT and PDT	[79]
		AuNPs	Sulfonated aluminum phthalocyanines conjugated	1064 nm, 39.9–420.1 W cm^−2^	SAS cells	Effective inactivation of oral cancer cells via combined PTT and PDT effects	[80]
		Au nanopopcorns	Stabilized with PEG through 11-mercaptoundecanoic acid and coated with silicon 2,3-naphthalocyanine dihydroxide	808 nm, 0.55 W cm^−2^	KB-3-1 cells, SK-BR-3 cells	First application of magnetic-field-guided drug delivery and dual-mode PTT/PDT using magnetic-optical hybrid nanosystems	[81]
		Cu_2−x_S	Magnetic manganese compounds integrated	808 nm, 0.72 W cm^−2^	HeLa cells, CDX mouse model, PDX mouse model	Reactive oxygen species (ROS) and heat generation enhance PTT, and O_2_ self-supplementation enhances PDT	[82]
		ICG, SWCNTs	ICG-conjugated hyaluronic acid nanoparticles encapsulated within SWCNTs	808 nm, 0.8 W cm^−2^	SCC 7 cells, CDX mouse model	CD44-targeted theranostic nanoparticle for PA image-guided dual PTT and PDT cancer therapy	[83]
		Cobalt-glycerate nanosheets	Folic acid modified	808 nm, 1.2 W cm^−2^	CAL 27 cells, residual OSCC tumors bearing mouse model, CDX mouse model	MRI-guided postsurgical PTT/PDT	[84]
		Au, Ce6	Polyethyleneimine functionalized with Au and Pt, followed by attachment of Ce6 and HN-1	1064 nm, 2 W cm^−2^, 650 nm, 0.5 W cm^−2^	SCC 9 cells, CDX mouse model	CT/FL/photothermal tri-modal imaging-guided treatment	[85]
	PTT, chemotherapy	ICG	Nanoparticles co-assembled from hydrophilic linear PEG and hydrophobic cholic acid cluster amphiphilic subunits	808 nm, 0.8 W cm^−2^	OSC 3 cells, orthotopic CDX mouse model, metastatic CDX mouse model	Versatile chemo-nanoplatform for synergistic PTT/chemotherapy of orthotopic oral cancer and immuno-nanoplatform for synergistic PTT/immunotherapy of metastatic cancer	[86]
			DOX-encapsulated PLGA nanoparticles with a cancer cell membrane and ICG surface coating	808 nm, 1.5 W cm^−2^	HSC 3 cells, CDX mouse model	Selective cancer cell targeting and induction of intrinsic mitochondria-mediated apoptosis via the p53 signaling pathway	[55]
		IR820	IR820/methylcellulose hydrogel containing mesoporous silica nanoparticles and DOX	808 nm, 2 W cm^−2^	CAL 27 cells, CDX mouse model	Long-term synergistic antitumor activity with lower toxicity	[87]
			IR820 and curcumin loaded onto hyaluronic acid microneedles	808 nm, 1 W cm^−2^	CAL 27 cells	Curcumin nanoparticles and IR820 microneedle combined drug delivery systems (DDS) have complete morphology and good mechanical properties	[88]
		AuNRs	Silica-coated AuNRs with a covalently assembled amphiphilic PLGA-PEG polymeric corona loaded with vincristine	808 nm, 1.2 W cm^−2^	SCC 15 cells, DMBA-induced HBP carcinoma	Coronabased drug delivery approach exhibited superior anticancer effects on OSCC	[89]
			Folate-targeted pegylated poly(D, L-lactide-co-glycolide) loaded with phytochemical anticancer thymoquinone and AuNRs	808 nm, 1.2 W cm^−2^	SCC 15 cells, DMBA-induced HBP carcinoma	Strong synergistic anticancer effects and selective tumor targeting via a dual-modal approach	[90]
		AuNPs	Endogenously double-controlled cisplatin prodrug incorporated	808 nm, 0.3 W cm^−2^	3D tumor models of SCC-25 and SCC-154	The first multifunctional nano-architecture (tNAscisPt) for combined chemotherapy and PTT	[91]
			PEG-stabilized and conjugated with PDPN antibody and DOX	532 nm, 1 W cm^−2^	CAL 27 cells, CDX mouse model	Versatile drug-delivery nanoplatforms for targeted and combined chemo-PTT against oral cancer	[92]
		GO	PEGylated GO linked with DOX and FAP-targeted peptide chains	808 nm, 1 W cm^−2^	CAL 27 cells, CDX mouse model	Precise targeting capability coupled with combined chemotherapy and PTT	[93]
			Modified with hyaluronic acid and HN-1 peptide and loaded with DOX	808 nm, 1 W cm^−2^	CAL 27 cells, CDX mouse model	Effective targeting of OSCC cells and outstanding localized deposition in xenograft tumors	[94]
			Loaded with ATP citrate lyase specific inhibitor (SB-204990) and DOX	808 nm, 1.5 W cm^−2^	SCC 15 cells, CDX mouse model	Synergistic treatment via lipid starvation, chemotherapy, and PTT	[95]
			GO and Cisplatin blended with poly(L-lactide) and hydroxypropyl methylcellulose	808 nm, 1.5 W cm^−2^	Human oral squamous cell carcinoma cell line UPCI-SCC-084, cisplatin-resistant cell line, 3D tumor spheroid model	3D-printed biodegradable implant with chemo-thermal ablation potential	[96]
		Cu (II)	Enveloped by chitosan	808 nm, 0.33 W cm^−2^	KB cells, CDX mouse model	Combined PTT and chemotherapy using CuCC NPs for noninvasive tumor ablation and reduced postoperative recurrence risk	[97]
			Polyethylene glycol-coated polyaniline NSs codoped with Cu (II) and vincristine	808 nm, 0.33 W cm^−2^	KBV cells, KB cells, CDX mouse model	MDR-tumor-targeted theranostics utilizing strong electrostatic interactions between resistant cells and nanomaterials	[98]
		Au nanoflowers	Two layers of silica coated	808 nm, 5–9 W cm^−2^	CAL 27 cells, HepG2 cells	Potentials for versatile loading and delivery of chemotherapeutic or photodynamic drugs	[99]
		Hollow mesoporous Prussian blue NPs (HMPBs)	DOX-loaded HMPBs within a hyaluronic acid microneedle system	808 nm, 2 W cm^−2^	CAL 27 cells, CDX mouse model	Thermal ablation and DOX release, promoted by the generated heat, induced apoptosis of tumor cells	[100]
		Chiral molybdenum (Cys-MoO_3−x_) NPs	Decorated with cysteine molecules	405–808 nm, 1 W cm^−2^	OSCC cells	Visible light and NIR dual-responsive properties for cancer cell ablation	[101]
		PDA	Functionalized with S-nitrosothiol, surrounded by gambogic acid-conjugated hyaluronic acid shells	808 nm, 1 W cm^−2^	HN6 cells, CDX mouse model	Tumor-selective nanocomplex for low-temperature photothermal therapy and NO-enhanced chemotherapy	[102]
	PTT, radiotherapy	AuNPs	Folate conjugated.	532 nm, 0.47 W cm^−2^	KB cells	Folate-conjugated gold nanoparticles induced cytotoxicity and cell apoptosis in KB cells	[103]
	PTT, gene therapy	AuNRs	Complexed with anionic-charged siRNA oligos	810 nm, 3.3 W cm^−2^	CAL 27 cells, CDX mouse model	Overcomes thermoresistance to sensitize cancer cells to hyperthermia	[104]
	PTT, immunotherapy	ICG	Gelatin nanoparticles loaded with ICG and NSC74859 (signal transducer activator of transcription 3, STAT3 inhibitor)	808 nm, 1 W cm^−2^	CAL 27 cells, CDX mouse model, HNSCC-bearing *Tgfbr1/Pten* 2cKO mouse model	Photothermal destruction of tumors combined with the STAT3 inhibitor elicited potent antitumor immunity for enhanced cancer therapy	[105]
		Organic photovoltaic material (PBDB-T NPs)	—	660 nm, 0.6 W cm^−2^	CAL 27 cells, CDX mouse model, immunocompetent and syngeneic mouse tumor model	Enhanced mPTT efficacy and active tumor-specific adaptive immune responses	[106]
		Molybdenum diphosphide nanorods (MoP_2_ NRs)	—	808 nm, 0.5 W cm^−2^	CAL 27 cells, SCC9 cells, CDX mouse model	Therapeutic modality via laser-potentiated peroxidase catalytic/mPTT	[107]
		Fe_3_O_4_ NPs	—	808 nm, 1 W cm^−2^	SCC 7 cells, CDX mouse model	Regulates the polarization of tumor-associated macrophages and enhances the inhibitory effect on tumor cells	[108]
		IR820	TAT peptide-conjugated IR820 incorporated into Poly(N-isopropylacrylamide) (PNIPAM)/demethylated lignin (DL) hydrogel	808 nm, 2 W cm^−2^	SCC 7 cells, bacteria-colonized tumor spheroids, bacteria-colonized tumor-bearing mouse, lung metastasis model with bacterial colonization	Photothermal ablation with robust stimulation of antitumor immune responses against bacteria-colonized OSCC	[109]
	PTT, PDT, chemotherapy	AuNPs	Combined with cisplatin-loaded BP NSs	808 nm	SCC 9 cells, DMBA-induced HBP carcinoma	High drug-loading capacity and excellent photothermal properties	[110]
		ICG	Coordination compound of ICG-cisplatin encapsulated into human serum albumin	808 nm, 2 W cm^−2^	HSC cells, CDX mouse model	Synergistic PTT/PDT/chemotherapy against OSCC via a NIR stimuli-responsive, tumor-targeted drug release system	[111]
		Coi8DFIC-sorafenib NPs	Coi8DFIC dye and sorafenib were used to construct CS NPs	808 nm, 0.5 W cm^−2^	CAL 27 cells, CDX mouse model	Laser on/off controlled vascular targeting therapy with CS NPs, guided by tumor-associated vessel tracking and real-time tumor imaging	[112]
		Pheophorbidea, Pa	Pa and DOX self-assembled, followed by the introduction of dual-aldehyde terminated polyethylene glycol2000 (PEG-2CHO)	680 nm, 0.4 W cm^−2^	OSC 3 cells, subcutaneous and orthotopic CDX tumor model	Dual size/charge-transformable Trojan-Horse nanoparticle for delivery of ultrasmall, full active pharmaceutical ingredients	[113]
	PTT, PDT, gene therapy	ICG	Poly(β-amino ester)/PLGA blended nanoparticles loaded with ICG, surface-adsorbed with Nrf2-siRNA, and encapsulated within cancer cell membrane vesicles	808 nm, 2 W cm^−2^	SCC 25 cells, CDX mouse model	Excellent PTT/PDT agent for OSCC treatment, where Nrf2-siRNA serves as an efficient photosensitizer synergist for PDT amplification	[114]
	PTT, PDT, immunotherapy	Ce6	Simvastatin (SIM)-packaged Ce6-PEG with a surface modification of targeting-antibody (anti-low-density lipoprotein receptor)	660 nm, 1 W cm^−2^	SCC 7 cells, homologous xenograft tumor mouse model	Cholesterol-regulating NPs with high tumor targeting and adjuvanticity for effective photo-induced immunotherapy	[115]
		IR780	Double-layered membrane vesicles extracted from attenuated *P. gingivalis* as an immune adjuvant	808 nm, 1 W cm^−2^	SCC 7 cells, CDX mouse model, metastatic CDX mouse model	Double-layered membrane vesicles derived from *P. gingivalis* applied as a novel bacterial adjuvant for activating antitumor immunity	[116]
		PDA	PDA-hyaluronic acid matrix loaded with protoporphyrin IX and aCD47-tagged CaCO_3_ NPs	808 nm, 1.2 W cm^−2^, 660 nm, 0.04 W cm^−2^	CAL 33 cells, low-immunogenic OSCC mouse model, residual tumor mouse model, orthotopic and subcutaneous CDX mouse model	Effectively prevents local recurrence, inhibits orthotopic OSCC growth and pulmonary metastasis, and provides long-term protective immunity against tumor rechallenge	[117]
Oral leu-koplakia (OLK)	PTT, PDT	ITIC-Th	—	660 nm, 1 W cm^−2^	CAL 27 cells, 4-nitroquinoline 1-oxide (4NQO)-induce oral leukoplakia mouse model	Effectively suppresses OLK cancerization without apparent topical or systemic toxicity and represents the first interdisciplinary research in multimodal therapy for OLK	[118]
	PTT, drug therapy	GO	Surface modification of GO with PEG via amide reaction, serving as a carrier to adsorb FAP targeting peptides and cyclooxygenase-2 inhibitors	808 nm, 1 W cm^−2^	DOK cells, 4-nitroquinoline 1-oxide (4NQO)-induced oral precancerous mice model	Nano-drug delivery system for targeting OLK with high FAP expression	[119]
	PTT, PDT, drug therapy	Mesoporous polydopamine nanoparticles	ICG and celecoxib co-loaded onto mesoporous polydopamine nanoparticles	808 nm, 1.5 W cm^−2^	DOK cells, 4-nitroquinoline 1-oxide (4NQO)-induced oral precancerous mice model	Transmucosal delivery of soluble microneedle-mediated integrated phototherapy anti-inflammatory NPs for OLK	[120]

Abbreviation. 3D: Three-dimensional; AuNPs: Gold nanoparticles; AuNRs: Gold nanorods; BP: Black Phosphorus; BSA: Bovine serum albumin; CDX: Cell line-derived xenograft; Ce6: Chlorin e6; CT: Computed tomography; DMBA: 7,12-Dimethylbenz[a]anthracene; DOX: Doxorubicin; EGFR: Epidermal growth factor receptor; FAP: Fibroblast activation protein; FL: Fluorescence; GO: Graphene oxide; HBP: Hamster buccal pouch; HNSCC: Head and neck squamous cell carcinoma; ICG: Indocyanine green; KBV: Human oral epithelial carcinoma vincristine-resistant tumor; mPTT: Mild-temperature photothermal therapy; MRI: Magnetic resonance imaging; NIR: Near-infrared; NSs: Nanosheets; OLK: Oral leukoplakia; OSCC: Oral squamous cell carcinoma; PA: Photoacoustic; PDA: Polydopamine; PDT: Photodynamic therapy; PDX: Patient-derived xenograft; *P. gingivalis*: *Porphyromonas gingivalis*; PLGA: Poly(lactic-co-glycolic acid); PLTs: Platelets; PTAs: Photothermal agents; PTT: Photothermal therapy; SWCNTs: Single-walled carbon nanotubes.

## Data Availability

Not applicable.

## Abbreviations

The following abbreviations are used in this manuscript:

3D	Three-dimensional
4NQO	4-nitroquinoline 1-oxide
APCs	Antigen-presenting cells
AuNPs	Au nanoparticles
AuNRs	Au nanorods
BMSCs	Bone marrow mesenchymal stem cells
BP	Black phosphorus
BSA	Bovine serum albumin
CDX	Cell line-derived xenograft
Ce6	Chlorin e6
COL	Collagen
CT	Computed tomography
CTAB	Cetyltrimethylammonium bromide
CTLs	Cytotoxic T lymphocytes
DMBA	7,12-dimethylbenz[a]anthracene
DOX	Doxorubicin
DDS	Drug delivery systems
EGFR	Epithelial growth factor receptor
EPR	Enhanced permeability and retention
FAP	Fibroblast activation protein
FL	Fluorescence imaging
GNR	Gold nanorod
GO	Graphene oxide
HA	Hyaluronic acid
HBP	Hamster buccal pouch
HMPBs	Hollow meso-porous Prussian blue NPs
HNSCC	Head and neck squamous cell carcinoma
HSP	Heat shock protein
HSR	Heat shock response
ICG	Indocyanine green
KBV	Human oral epithelial carcinoma vincristine-resistant tumor
LSPR	Localized surface plasmon resonance
MMP	Matrix metalloproteinase
mPTT	Mild-temperature photothermal therapy
MRI	Magnetic resonance imaging
nHA	Nanohydroxyapatite
NIR	Near-infrared
NPs	Nanoparticles
NSs	Nanosheets
OLK	Oral leukoplakia
OPMDs	Oral potentially malignant disorders
OSCC	Oral squamous cell carcinoma
PA	Photoacoustic
PDA	Polydopamine
PDT	Photodynamic therapy
PEG	Poly(ethylene glycol)
PLGA	Poly(lactic-co-glycolic acid)
PLTs	Platelets
PPTT	Plasmonic photothermal therapy
PTT	Photothermal therapy
*P. gingivalis*	*Porphyromonas gingivalis*
PS	Photosensitizers
PT	Photothermal
PTAs	Photothermal agents
PTT	Photothermal therapy
RB	Rose bengal
ROS	Reactive oxygen species
RT	Radiotherapy
SF	Silk fibroin
SIM	Simvastatin
siRNA	Small interfering RNA
STAT3	Signal transducer activator of transcription 3
SWCNTs	Single-walled carbon nanotubes
TME	Tumor microenvironment
VCR	Vincristine
